# Hidden Threads: Unusual Aortic Dissection Manifesting Through Abdominal Pain

**DOI:** 10.7759/cureus.90650

**Published:** 2025-08-21

**Authors:** Priscila Lopez, Nicole Del Orbe, Sankeerth Thoota, Allah Yar, Damian Kurian

**Affiliations:** 1 Internal Medicine, Harlem Hospital Center, New York, USA; 2 Internal Medicine/Cardiology, Harlem Hospital Center, New York, USA

**Keywords:** aortic dissection, epigastric pain, hypertensive emergency, stanford classification, vascular

## Abstract

Aortic dissection is an uncommon but life-threatening condition with a wide range of clinical presentations that can mimic other pathologies, often complicating timely diagnosis. Management strategies vary depending on the dissection type and clinical context. We report the case of a 34-year-old male with a history of uncontrolled hypertension with medication non-adherence who presented with dyspnea and abdominal pain after smoking marijuana. He was initially treated for flash pulmonary edema in the setting of a hypertensive emergency. However, due to persistence and worsening symptoms despite initial management, a CT of the chest was performed, revealing type A aortic dissection. The patient subsequently underwent emergent vascular surgery.

## Introduction

Aortic dissection, though relatively uncommon, is a catastrophic illness demanding prompt and precise diagnosis for patient survival. With an estimated incidence of 2-3.5 per 100,000 persons per year, acute aortic dissection poses a significant threat. The mortality rate escalates by 1% to 2% per hour without intervention, reinforcing the importance of a fast and accurate diagnosis [1]. The most significant challenge associated with aortic dissection is its ability to mimic other more common conditions, such as myocardial ischemia, exacerbation of heart failure/chronic obstructive pulmonary disease (COPD), pulmonary embolism, or gastrointestinal illness [2].

The typical presenting symptoms of the condition often include chest or back pain, syncope, and shortness of breath; however, the rare and atypical symptoms must not be overlooked as it can delay the diagnosis, especially in non-pain-related symptoms [2] Point-of-care ultrasound (POCUS) plays a vital role in rapidly diagnosing aortic dissection at the bedside, especially in patients who are hemodynamically unstable and in whom CT angiography cannot be performed [3]. Despite the challenges encountered in diagnosing aortic dissection, early diagnosis is imperative as the mortality rate for untreated patients can increase to 1% per hour once the symptoms begin [4]. We highlight a rare presentation of aortic dissection in a male patient that commenced after marijuana use and presented with abdominal pain and shortness of breath. Although the role of marijuana is understudied in this context, it may be considered a risk factor for aortic dissection, especially in young patients with other comorbidities [5].

## Case presentation

A 34-year-old male with a significant past medical history presented to the emergency department (ED) with a sudden-onset shortness of breath and epigastric abdominal pain. His medical history included a remote left thoracoabdominal stab wound requiring exploratory laparotomy and tube thoracostomy for a left-sided hemopneumothorax, poorly controlled hypertension due to medication non-adherence, and a previous episode of pneumonia complicated by empyema, for which he had undergone right thoracotomy with decortication and wedge resection one year prior. That hospitalization had been complicated by acute kidney injury secondary to acute tubular necrosis, requiring continuous renal replacement therapy.

The patient reported that symptoms had begun shortly after smoking marijuana. He endorsed subjective fevers for three days and orthopnea (requiring two to three pillows), but denied chest pain, palpitations, dizziness, headaches, leg swelling, or decreased exercise tolerance. Emergency Medical Services (EMS) documented a blood pressure of 261/130 mmHg, for which sublingual nitroglycerin was administered and continuous positive airway pressure (CPAP) initiated en route to the hospital. Upon arrival to the ED, his vital signs were notable for a blood pressure of 195/119 mmHg, heart rate of 140 bpm, respiratory rate of 40 breaths per minute, and temperature of 97 °F. Physical examination revealed a patient in significant respiratory distress with bilateral crackles on pulmonary auscultation. Surgical scars were noted on the chest and abdomen; the remainder of the physical exam was unremarkable.

Laboratory evaluation revealed a high anion gap metabolic acidosis due to lactic acidosis with concurrent respiratory acidosis. High-sensitivity troponin level was 24 ng/L, and proBNP was 7,779 pg/mL. Urine toxicology screening, including tests for cocaine, barbiturates, opiates, benzodiazepines, and methadone, was negative, and the rest was unremarkable (Table 1).

**Table 1:** Laboratory testing results WBC: white blood cells; HGB: hemoglobin; HCT: hematocrit; PCO_2_: partial pressure of carbon dioxide; TCO_2_: total carbon dioxide; PO_2_: partial pressure of oxygen; HCO_3_: bicarbonate; HS: highly sensitive; ProBNP: pro-B-type natriuretic peptide

Component	Results - on admission	Results - a few hours later		Reference range and units
WBC	7.73	14.19	19.56	4.80 - 10.80 x 10^3^/mcL
HGB	14.2	14.8	14.1	14.0 - 18.0 g/dL
HCT	44.9	45.5	41.5	42.0 - 52.0%
Platelets	227	247	222	150 - 450 x 10^3^/mcL
BUN	13	18	26	5 - 26 mg/dL
Creatinine	1.1	1.2	1.3	0.70 - 1.20 mg/dL
PH venous	7.09	7.24	7.21	7.32 - 7.43
PCO_2_ venous	61	55	57	41 - 54 mmHg
Lactate	8.4	5.7	6.4	0.6 - 1.4 mmol/L
TCO_2_ venous	20	25	24	22 - 26 mmol/L
PO_2_ venous	38	37	38	30 - 50 mmHg
HCO_3_ venous	18	24	22	22 - 29 mmol/L
Troponin T HS	24	83		0 - 22 ng/L
ProBNP	7,779	14,588	27,889	≤125 pg/mL
Alcohol	<10			≤10 mg/dL
Barbituates urine	Negative			Negative
Benzodiazepine urine	Negative			Negative
Cocaine urine	Negative			Negative
Opiates urine	Negative			Negative

**Figure 1:**
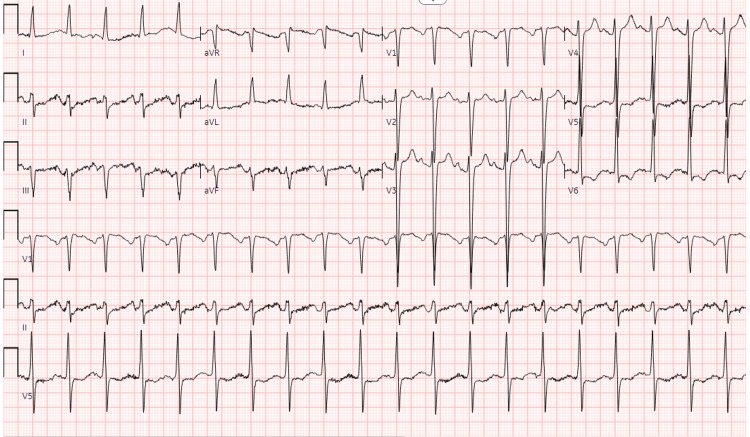
Electrocardiogram #1 The findings show sinus tachycardia (heart rate 120); left axis deviation; left anterior fascicular block; and LVH with repolarization abnormalities LVH: left ventricular hypertrophy

Electrocardiography showed sinus tachycardia with left axis deviation, left anterior fascicular block, and left ventricular hypertrophy (LVH) with repolarization abnormalities (Figure 1).

Initial management included nitroglycerin infusion, which reduced blood pressure to 170/100 mmHg before being discontinued. Furosemide was administered, and the patient was placed on bilevel positive airway pressure (BiPAP) per the recommendation of the cardiac critical care consult team. He was admitted to the general medical floor with a diagnosis of hypertensive emergency and flash pulmonary edema, with consideration for possible heart failure. Medical therapy with furosemide, metoprolol, and amlodipine was initiated. Despite these interventions, the patient’s respiratory distress persisted, and blood pressure remained elevated.

**Figure 2:**
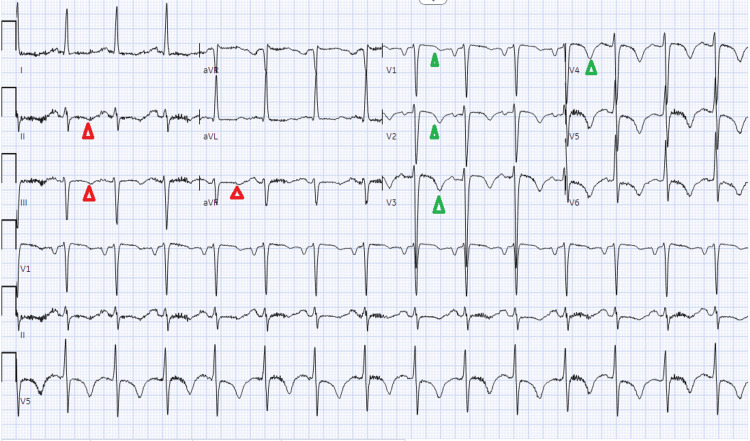
Electrocardiogram #2 The findings show a normal sinus rhythm, left atrial enlargement, left anterior fascicular block, LVH (R in aVL, Cornell product), and prolonged QT (583 ms). When compared with EKG #1, inverted T waves have replaced nonspecific T wave abnormality in inferior leads (red arrowheads). T wave inversion is now evident in the anterior leads (green arrowheads) LVH: left ventricular hypertrophy

Approximately 12 hours after admission, the patient experienced worsening dyspnea, adopted a tripod position, and developed tachypnea (respiratory rate ~40 breaths/min), profuse diaphoresis, and cool extremities-suggestive of impending respiratory failure. He was transferred to the medical ICU (MICU). At that time, he reported localized epigastric and retroxiphoid pain. Morphine was administered with mild relief, with safety monitoring measures in place. Noninvasive ventilation was transitioned to CPAP, and a nitroglycerin drip was reinitiated. Repeat ECG demonstrated normal sinus rhythm, left atrial enlargement, persistent left anterior fascicular block, LVH, prolonged QT interval, and evolving T wave changes-specifically T wave inversions in anterior leads and dynamic changes in inferior leads (Figure 2). Transthoracic echocardiography revealed severe LVH, mild right ventricular hypertrophy, impaired diastolic relaxation, biatrial enlargement, moderate aortic insufficiency, and a mildly dilated aortic root with limited visualization-raising suspicion for aortic dissection.

**Figure 3:**
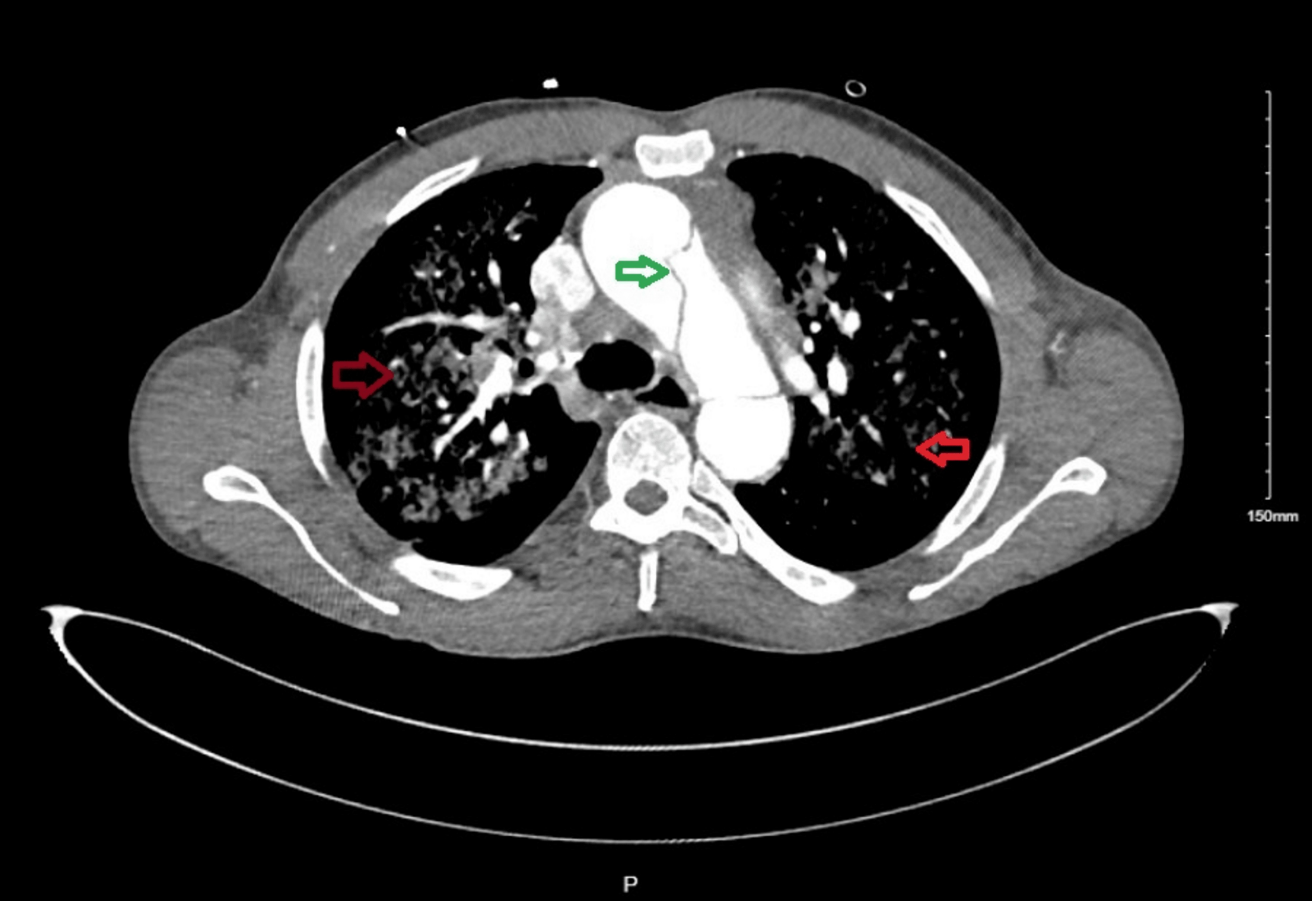
CTA of the chest - transverse plane Green arrow - acute type A aortic dissection involving the ascending aorta extending into the left subclavian artery and descending aorta. Red arrows - extensive ground glass opacities present in all lobes of the lungs CTA: computed tomography angiography

**Figure 4:**
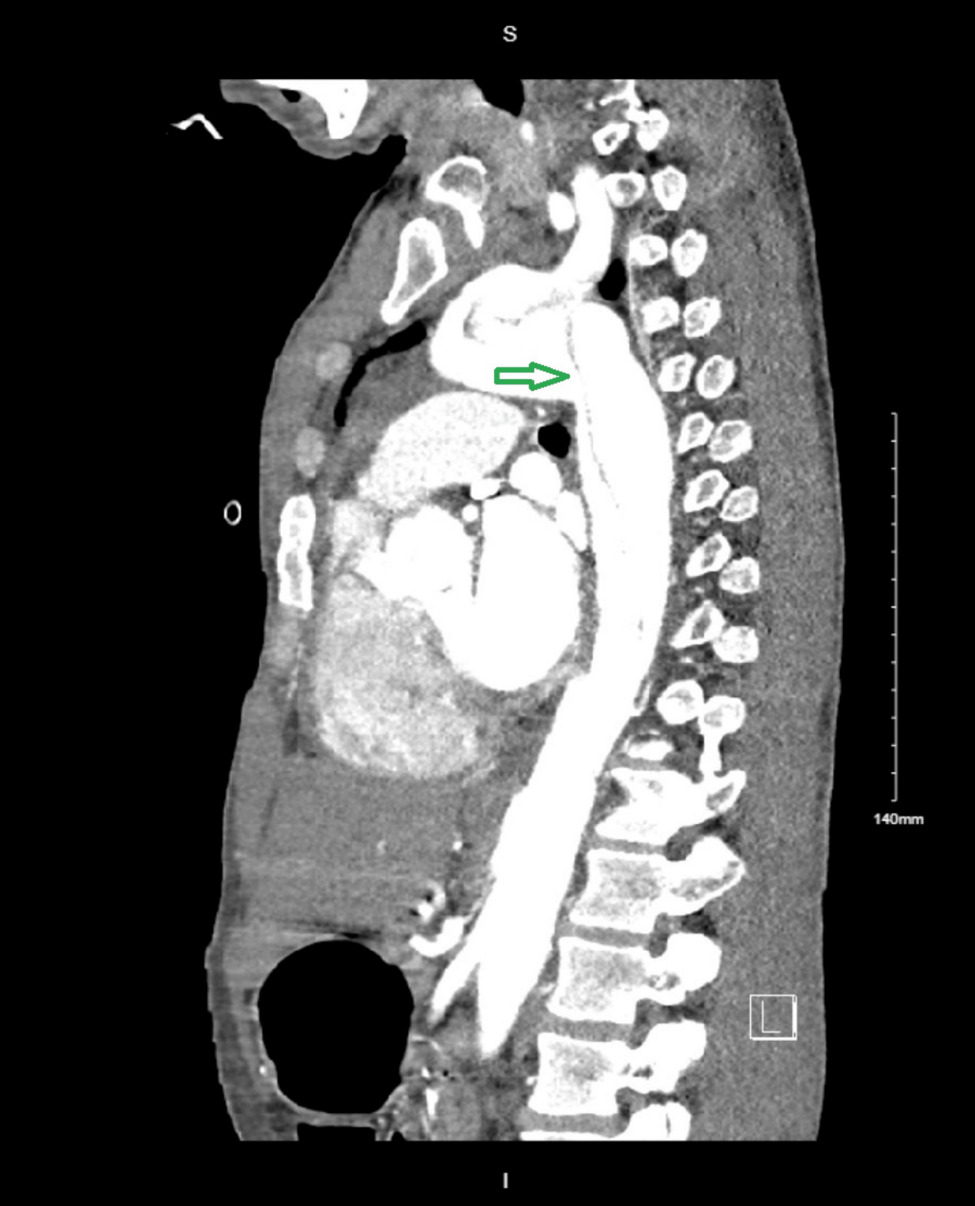
CTA of the chest - sagittal plane Green arrow: acute type A aortic dissection involving the descending aorta CTA: computed tomography angiography

A CT angiography CTA of the chest confirmed an acute Stanford type A aortic dissection involving the ascending aorta, with extension into the left subclavian artery and descending aorta. Extensive bilateral ground-glass opacities were also noted (Figures 3, 4).

The patient was started on intravenous labetalol and transferred to a tertiary care center approximately 20 hours after the initial presentation for emergent cardiovascular surgery. Intraoperatively, chronic dissection and severe aortic insufficiency were identified. The patient underwent successful surgical repair with hemiarch replacement and aortic valve resuspension.

Postoperatively, the patient was managed in the ICU with dobutamine, nicardipine, and continuous furosemide infusion. Once clinically stable, he was transitioned to an oral medication regimen including furosemide 20 mg daily, aspirin 81 mg daily, amlodipine 10 mg daily, labetalol 600 mg three times daily, and hydralazine 25 mg three times daily. The patient was discharged home in stable condition with cardiothoracic surgery follow-up and a scheduled repeat imaging evaluation in three months.

## Discussion

Aortic dissection is characterized by a tear in the intimal layer of the aortic wall, allowing blood to enter the medial layer and creating a separation between the layers. This process results in the formation of a true lumen and a false lumen, which may or may not communicate with each other [6]. According to the Oxford Vascular Study, the incidence of aortic dissection is approximately six per 100,000 persons per year, with a mortality rate reaching 50% within 48 hours in the absence of surgical intervention [7]. Aortic dissection is a highly lethal condition, notable for its sudden onset and severe clinical consequences. Although it typically presents with acute chest or back pain [4], our case involved a 34-year-old male who initially presented with abdominal pain and dyspnea, symptoms initially misattributed to acute decompensated heart failure. This report underscores the diagnostic challenge in differentiating aortic dissection from more common conditions, such as myocardial ischemia or gastrointestinal disorders, particularly when symptoms are atypical.

While classic presentations include chest or back pain, syncope, and shortness of breath [4], this case highlights the importance of recognizing that nonspecific or atypical symptoms-such as epigastric abdominal pain-can also indicate an underlying aortic dissection. It emphasizes the critical need for clinicians to maintain a high index of suspicion and include aortic pathology in the differential diagnosis, even in the absence of hallmark features. Although stimulants, tobacco, and alcohol are the more common substances that can cause aortic dissection, marijuana has been considered a risk factor as well, especially in young patients with underlying comorbidities [5]. Our patient was found to have chronic aortic dissection, which correlates with his non-adherence to medication, which possibly predisposed him to develop a pre-existing and less severe dissection that acutely worsened, triggered by the hypertensive crisis.

Classification systems, such as the Stanford system (type A involving the ascending aorta, and type B involving the descending aorta) and the DeBakey classification (type I involving the ascending aorta and arch, type II limited to the ascending aorta, and type III originating in the descending aorta), provide a structured framework for understanding the anatomical extent and clinical implications of aortic dissections. The current case is categorized as an acute Stanford type A dissection, with involvement of the ascending aorta, left subclavian artery, and descending aorta [8].

The management of aortic dissection requires a prompt and nuanced approach, with early recognition and intervention being critical to improving patient outcomes [9]. Initial medical management includes pain control, administration of short-acting intravenous beta-blockers, and strict blood pressure control to reduce shear stress on the aortic wall. While medical therapy is often sufficient for uncomplicated type B dissections, type A dissections typically require emergent surgical intervention [10,11]. This case underscores the importance of timely diagnosis and the urgent nature of intervention, especially in the context of atypical clinical presentations. Within 24 hours, our patient was diagnosed and underwent successful cardiothoracic surgery, leading to a favorable outcome. Despite diagnostic challenges, the use of advanced imaging modalities and adherence to established clinical guidelines remain essential for accurate diagnosis and optimal management.

## Conclusions

This report underscores the complexity and diagnostic challenges of cardiovascular emergencies, particularly in patients with poorly controlled hypertension and medication non-adherence. The initial presentation with hypertensive crisis and flash pulmonary edema obscured the underlying, life-threatening pathology: an acute Stanford type A aortic dissection. The patient's clinical deterioration despite appropriate initial management prompted advanced imaging, ultimately revealing the diagnosis. This report highlights the critical importance of maintaining a broad differential diagnosis in patients with persistent or atypical symptoms, even when an initial explanation seems plausible. Aortic dissection, though uncommon, must be considered in hypertensive patients presenting with chest, back, or unexplained abdominal pain, and the use of marijuana should be considered a risk factor in young patients. Early recognition, supported by appropriate diagnostic imaging and clinical suspicion, is essential to reduce morbidity and mortality. Heightened vigilance, timely evaluation, and adherence to evidence-based management strategies remain paramount in improving outcomes in such high-risk presentations.

## References

[1] Harris KM, Nienaber CA, Peterson MD (2022). Early mortality in type A acute aortic dissection: insights from the International Registry of Acute Aortic Dissection. JAMA Cardiol.

[2] Kurabayashi M, Miwa N, Ueshima D (2011). Factors leading to failure to diagnose acute aortic dissection in the emergency room. J Cardiol.

[3] Bunce C, Bryczkowski C, Rometti M (2023). Aortic dissection case report. J Educ Teach Emerg Med.

[4] Levy D, Sharma S, Farci F, Le JK (2025). Aortic Dissection. https://www.ncbi.nlm.nih.gov/books/NBK441963/.

[5] Sarmiento IC, Giammarino A, Scheinerman SJ, Guirola A, Hartman A, Brinster D, Hemli JM (2021). Marijuana: an underappreciated risk factor for acute type A aortic dissection?. Heart Surg Forum.

[6] Telci O, Ozkok A, Tekce G, Oguz A (2012). Aortic dissection presenting with abdominal pain. Intern Med.

[7] Sayed A, Munir M, Bahbah EI (2021). Aortic dissection: a review of the pathophysiology, management and prospective advances. Curr Cardiol Rev.

[8] Howard DP, Banerjee A, Fairhead JF, Perkins J, Silver LE, Rothwell PM (2013). Population-based study of incidence and outcome of acute aortic dissection and premorbid risk factor control: 10-year results from the Oxford Vascular Study. Circulation.

[9] Yu HC, Wang ZQ, Hao YY (2015). An extensive DeBakey type IIIb aortic dissection with massive right pleural effusion presenting as abdominal pain and acute anemia: particular case report. J Geriatr Cardiol.

[10] Orihashi K (2012). Acute type a aortic dissection: for further improvement of outcomes. Ann Vasc Dis.

[11] Wong DR, Lemaire SA, Coselli JS (2008). Managing dissections of the thoracic aorta. Am Surg.

